# Use or Consequences: Probing the Cognitive Difference Between Two Measures of Divergent Thinking

**DOI:** 10.3389/fpsyg.2018.02327

**Published:** 2018-11-27

**Authors:** Richard W. Hass, Roger E. Beaty

**Affiliations:** ^1^College of Humanities and Sciences, Thomas Jefferson University-East Falls, Philadelphia, PA, United States; ^2^Department of Psychology, Pennsylvania State University, University Park, PA, United States

**Keywords:** creativity, divergent thinking, memory search, default mode network, semantic memory

## Abstract

Recent studies have highlighted both similarities and differences between the cognitive processing that underpins memory retrieval and that which underpins creative thinking. To date, studies have focused more heavily on the Alternative Uses task, but fewer studies have investigated the processing underpinning other idea generation tasks. This study examines both Alternative Uses and Consequences idea generation with a methods pulled from cognitive psychology, and a novel method for evaluating the creativity of such responses. Participants were recruited from Amazon Mechanical Turk using a custom interface allowing for requisite experimental control. Results showed that both Alternative Uses and Consequences generation are well approximated by an exponential cumulative response time model, consistent with studies of memory retrieval. Participants were also slower to generate their first consequence compared with first responses to Alternative Uses, but inter-response time was negatively related to pairwise similarity on both tasks. Finally, the serial order effect is exhibited for both tasks, with Consequences earning more creative evaluations than Uses. The results have implications for burgeoning neuroscience research on creative thinking, and suggestions are made for future areas of inquiry. In addition, the experimental apparatus described provides an equitable way for researchers to obtain good quality cognitive data for divergent thinking tasks.

## 1. Introduction

Creative thinking studies have long depended on classic divergent thinking tasks as operationalizations of the construct. With recent emergence of studies using a variety of neuroimaging techniques to examine the cognitive roots of performance on the tasks, there became a need for more probing cognitive analyses of divergent thinking. To some extent, this has been done (Beaty et al., 2014; Forthmann et al., 2016; Acar and Runco, 2017; Hass, 2017a) but such analyses have focused almost exclusively on responses to the Alternative Uses task, in which participants are asked to generate as many creative uses for common objects as possible within a specified time period (usually 2–3 min).

This study was motivated by several perceived gaps in both methodology and theory of another oft-used divergent thinking task: the Consequences task (Wilson et al., 1954; Torrance, 1974). First, when participants generate responses to prompts from the consequences task (e.g., “imagine that humans no longer needed to sleep”), it is not altogether clear whether the idea generation process unfolds in a similar fashion to idea generation to Alternative Uses prompts (e.g., “think of creative uses for a brick”). Second, it is somewhat more difficult for judges to agree on creativity ratings assigned to consequences responses (Silvia et al., 2008; Hass et al., 2018). Indeed, it seems that scoring consequences tasks involves an increase in cognitive load over the scoring of alternative uses tasks (Forthmann et al., 2017). Finally, though other researchers are beginning to examine response time distributions as evidence of cognitive processing during divergent thinking (Acar and Runco, 2017), the full scope of analyses that can be done with response times has not fully been explicated.

The novel components of this study follow from the points just raised. In this paper we present a web-based data collection methodology for divergent thinking tasks (and indeed any kind of creative thinking task that urges multiple responses), which was designed using the tools created by the psiTurk group (McDonnell et al., 2012). There are indeed other methods by which one can use electronic means of collecting DT data, but the importance of this web-based tool is that psiTurk was designed specifically to allow researchers to collect precise cognitive data from workers on the Amazon Mechanical Turk platform (MTurk). As such, it allows for researchers of all levels to easily collect creative thinking data from a more representative sample than is often available on university campuses. In addition, we illustrate how cognitive theory can be applied to response time data culled from both Alternative Uses and Consequences tasks. Finally, we use a newly validated scale for measuring creativity of responses, along with human rated similarity of responses to compare and contrast the response generation process across these two tasks.

### 1.1. Divergent thinking and memory processes

The study of divergent thinking in general has spanned generations of creativity researchers. Though the tasks that measure divergent thinking are disparate (e.g., Forthmann et al., 2018), and may not be interchangeable (cf. Silvia, 2011; Runco et al., 2016), this study was focused on cognitive analyses of the acts of generating alternative uses for objects, and generating consequences of impossible situations. Specifically, the central question of this analyses was whether or not the memory processes involved in generating these two types of divergent thinking responses overlap, or are distinct. Do answer that question, methods culled from the cognitive science of memory recall were used in conjunction with methods from the creativity literature. This section summarizes the relevant aspects of the cognitive science of memory recall.

Several past results in the literature on memory retrieval provide a context for the current study. First, in one of the foundational studies on divergent thinking, Christensen et al. (1957) plotted the number cumulative responses to various cues as a function of time elapsed. Along with alternative uses cues, the authors plotted results from the classic Bousfield and Sedgewick (1944) study of cumulative responding and semantic memory retrieval, in which they derived the well known negative exponential function which describes the decreasing output rate for generating category exemplars like fruits and animals. Wixted and Rohrer (1994) reviewed the results of subsequent studies concluding that the function is evidence of a repeated sampling of semantic space during memory retrieval, which is then depleted leading to more false retrievals, and an exponential slowing of retrieval rate (see also Raaijmakers and Shiffrin, 1981). Hass (2017a) found similar exponential slowing of response rates when participants generated uses for objects, but also found that generating uses yields lower response totals, and that response arrays were looser in terms of pairwise semantic relationships (cf. Troyer et al., 1997).

The Christensen et al. (1957) study on divergent thinking provided more direct evidence of differences, not only between creative idea generation and memory retrieval, but among different idea generation prompts. First, the output totals for divergent thinking cues were among the lowest reported (the lowest being output totals for words containing the letters M, T, or D, a very constraining memory retrieval task). Second, the cumulative response curves for two divergent thinking prompts: alternative uses for a brick, and impossibilities (“think of all of the impossible things”), were more linear than those for classic memory retrieval cues (e.g., U.S. cities). The “impossibilities” prompt is similar to the more common “consequences” prompt, with the latter simply specifying an impossibility of which participants generate consequences, while the former involves participants generating impossibilities with no specific context. So on that basis, there may be little difference in cumulative output when comparing alternative uses prompts to consequences prompts. However, in the Christensen and colleagues study, participants generated ideas for over 10 min, and the cumulative response functions were plotted across 2 min blocks. It may be that a more granular analysis of cumulative responding will yield subtle differences in the output functions when alternative uses and consequences prompts are compared. Indeed, there is reason to believe that differences should exist between the two tasks, and the argument forwarded presently is that alternative uses responding and consequences responding may rely on different contributions of episodic and semantic memory and also reasoning.

#### 1.1.1. Episodic and semantic memory and divergent thinking

Much of the existing work on characterizing the contributions of memory retrieval to divergent thinking has focused on semantic memory (e.g., Gilhooly et al., 2007; Abraham and Bubic, 2015; Kenett et al., 2016; Hass, 2017a,b). One line of research has examined how individual differences in semantic retrieval ability (i.e., verbal fluency or “broad retrieval ability”) relates to divergent thinking fluency and originality. Silvia et al. (2013) administered a battery of verbal fluency tasks, corresponding to lower-order facets of retrieval ability (e.g., associational fluency; listing as many words in a given category as possible), and found that a higher-order “retrieval ability” factor comprised of the lower-order factors strongly predicted the quantity and quality of ideas generated on the Alternative Uses task, suggesting that the general ability to fluently retrieve a range of concepts from semantic memory is central to verbal divergent thinking performance (see also Benedek et al., 2012; Avitia and Kaufman, 2014). Subsequent work has found that both controlled access to semantic memory (via verbal fluency) and the underlying structure of semantic concepts in memory contribute to divergent thinking (Beaty et al., 2014; Benedek et al., 2017), lending support to so-called “dual-process” models of creative cognition that emphasize the involvement of both top-down (executive) and bottom-up (associative) processes (Barr et al., 2015; Sowden et al., 2015).

Aside from the contributions of broad retrieval abilities, recent analyses of divergent thinking using network analysis (Kenett et al., 2014) and response time analysis (Hass, 2017a) have illustrated that the structure of semantic memory influences divergent thinking responding (see also Forthmann et al., 2016). Kenett and colleagues showed that a more “flexible” semantic network structure relates to high-divergent thinking ability and self-reported creative achievement (Kenett et al., 2016), likely reflecting an organization of semantic memory that is more conducive to establishing more remote conceptual links. Building off of Hass's work, Xu (2017) further showed that when constraining participants to only think of “new” ideas during alternative uses responding, the response time functions were more linear, yielding higher predicted output totals, and higher originality, compared to phases in which participants were instructed to think of “old” ideas. Finally, there seems to be a robust serial order effect in alternative uses responses such that early responses earn lower creativity ratings than responses generated later in the responding interval (Christensen et al., 1957; Beaty and Silvia, 2012; Hass, 2017b; Wang et al., 2017).

A recent verbal protocol analyses of divergent thinking, Gilhooly et al. (2007) showed that the retrieval of known uses for objects from episodic memory dominates initial alternative uses responding. That result provides an explanation for the serial order effect such that known object uses should be rated as less creative than uses created on the spot by participants. The results presented by Gilhooly and colleagues also spurred a number of studies designed to test whether an “episodic specificity induction”, an exercise where participants are trained to retrieve details from “recent experiences”, affected the fluency and flexibility of divergent thinking responding (e.g., Madore et al., 2015, 2016, 2017). Madore et al. (2015) showed that the induction enhanced the number of categories of uses (also known as flexibility) during divergent thinking, but did not enhance the number of objects generated in an association task. Similarly, Madore et al. (2016) showed that the induction enhanced responding on both an alternative uses and a consequences task. However, in the latter study, the effects were constrained to counts of participant-rated “old” vs. “new” responses (following Benedek et al., 2014). Madore et al. (2016) pointed out that the participants reported generating many more “new” responses on the consequences task, leading them to conclude that the task relies less on recalling specific episodes compared with alternative uses responding.

This characterization is also in line with increasing evidence from functional brain imaging research. Several functional MRI studies have reported activation within a set of brain regions collectively known as the default network (DN) when participants are engaged in creative thinking tasks in the scanner. The DN shows robust engagement during episodic memory retrieval and episodic future simulation tasks, which require the flexible recombination of episodic content (e.g., people, places, and actions) to reconstruct past experiences and imagine possible future experiences (Buckner et al., 2008). As noted above, Madore et al. (2015) have shown that an episodic specificity induction selectively enhances performance on the AUT, potentially reflecting the involvement of constructive episodic retrieval mechanisms (Schacter and Madore, 2016).

A recent fMRI study involved administering the episodic induction in the scanner and found that the induction was associated with increased divergent thinking performance, which corresponded to increased activity within the left anterior hippocampus (Madore et al., 2017), a region within the DN involved in episodic simulation. Several other studies have reported functional connectivity (i.e., correlation in neural responses) between regions of the DN and regions involved in cognitive control associated with creative task performance (Green et al., 2015; Beaty et al., 2017a,b, 2018; Gao et al., 2017; Zhu et al., 2017; Bendetowicz et al., 2018; Chen et al., 2018; Shi et al., 2018; Sun et al., 2018; Vartanian et al., 2018). Cooperation between DN and control regions is thought to reflect an interplay between idea generation and idea evaluation, retrieving possible solutions from memory and modifying them to fit task constraints (Beaty et al., 2016; Beaty and Schacter, 2018).

### 1.2. Differentiating uses and consequences tasks

The points raised in the preceding discussion lean heavily on the use of the Alternative Uses task as the proxy measure of creative thinking (but see Addis et al., 2016). Given that discussion, it seems clear that Alternative Uses responding begins with a memory search process similar to the search that unfolds when people generate members of a well learned category. However, as responding continues, people rely less on known instances of an object's use, and begin to exploit properties of objects to discover new uses via some sort of simulation process. Individual differences in the ability to generate creative uses has been tied both to fluid intelligence and to functional connectivity between cognitive control brain regions and memory-related regions within the DN. However, it is unclear if these conclusions extend to idea generation when the Consequences task is used as the proxy measure of creative thinking.

As mentioned, Madore et al. (2016) provided evidence that the reliance on episodic memory retrieval is weaker for the consequences task, but that result requires further investigation. There are other mechanisms that may be at work during consequences responding beyond memory retrieval and episodic simulation. To name one, the consequences task may require a form of counterfactual reasoning (Byrne, 2002; Abraham and Bubic, 2015) such that participants must consider what would happen if an enduring property of the world changed (e.g., gravity ceased to exist). However, when cognitive psychologists study counterfactual reasoning, the experimental methods often require participants to learn about novel situations and then create counterfactuals using reasoning (e.g., about placing bets Dixon and Byrne, 2011). Analysis usually focuses on how participants' reasoning changes based on information contained in the description of the event (e.g., contrasting “normal” behavior of the agent with “extraordinary” behavior). In those studies, counterfactual reasoning is a given, and the goal is to discover how context influences the course of reasoning. In the consequences task specifies, a participant is supplied with a counterfactual antecedent (*if humans no longer need sleep*) and must then supply as many consequences (*then humans will not need to do X*) as possible (Forthmann et al., 2017). The goal of most studies using consequences tasks is simply to provide a proxy of creative thinking that can be correlated with other variables, or contrasted across groups. Thus, it may be difficult to ascertain whether or not counterfactual reasoning is at work during consequences responding. Still, the general hypothesis that can be tested currently is that consequences generation entails a lengthier processes compared with uses generation due primarily to additional reasoning that might be required.

The aim of this study was to use response time models, human rated semantic similarity, and a newly validated rating scale for DT responses to attempt to distinguish the course of alternative uses responding from consequences responding. There are three distinct predictions that follow from such analyses and the information reviewed in previous sections. First, the serial order effect for alternative uses responding seems to be a function of the early reliance on episodic retrieval and then the continued use of episodic and semantic simulation to derive more and more remote associations between known properties of objects and novel uses for those objects. Given that the consequences task often results in “new” responses, which may indicate that episodic memory is less of a factor, we hypothesize that the serial order effect should either be flatter for consequences responses. That is, it may be that when responding to consequences items, instead of searching quickly for a specific episode (which indeed seems impossible) participants instead arrive at a consequence through some type of reasoning (possibly counterfactual reasoning). For example, if given the prompt to think of [creative] consequences that would result if humans no longer needed sleep, a participant might search for knowledge related to sleep, and then use counterfactual reasoning to derive successive consequences (i.e., consider what might [not] happen if those facts about sleep became false). This, in turn, would yield a potentially more creative response earlier in the response sequence, thus affecting the rate of change in the relationship between the order of responding and creativity (Prediction 1).

The second predicted difference between consequences and alternative uses responding is in the dynamics of response times. There are two sub-predictions here. First, if it is the case that consequences responding is not a simple function of memory search (i.e., involves counterfactual reasoning, or some other process), the initial response time for a consequences prompt should be slower than the initial response time for alternative uses. Previous analyses suggest that on average people take between 2 and 4 s to generate their first use in an alternative uses task (Hass, 2017a). Theories of semantic memory search suggest that this initial response latency is a function of the initial encoding of the cue, and the initialization of search processes (Wixted and Rohrer, 1994). If this initial encoding for consequences responding also involves counterfactual reasoning (or other processes), then the latency to the first response should be longer. Second, if it is the case that the consequences responding continually requires new creation of counterfactual consequences, the rate of responding should also be affected. As reviewed, Hass (2017a) showed that alternative uses responding is consistent with the negative exponential rate of search that is typical of semantic memory search. Explanations for the negative exponential rate usually center around the fact that semantic memory is a finite store and repeated search and recall of information will lead to a depletion of to-be-recalled information, exponentially slowing search. If it is the case that consequences responding does not simply involve search and retrieval from episodic and semantic stores, then a negative exponential function is not likely to fit response times. This also follows from the analysis by Xu (2017), which showed that when participants are constrained to only generating “new” alternative uses responses (i.e., avoiding the initial reliance on episodic stores), the cumulative response function appears more linear than exponential. More specifically, the rate of the cumulative response function is slower in the latter case. Since Madore et al. (2016) demonstrated that consequences response arrays are dominated by “new” responses, then consequences response curves should also be more linear than alternative uses response curves.

The final prediction tested in this analysis involves the semantic similarity of successive responses. In ordinary memory search using free-recall paradigms (e.g., naming all the animals one knows), participants often generate clusters of similar responses in short succession (e.g., farm animals such as cow, pig, goat, etc.). Several explanations for the phenomenon exist that are out of the scope of the current paper (cf. Gruenewald and Lockhead, 1980; Herrmann and Pearle, 1981; Troyer et al., 1997; Hills et al., 2012; Abbott et al., 2015), but generally pertain to the question of whether memory itself is a clustered representation, and/or whether search processes exploit certain features of the memory store. Hass (2017a) showed that clustering is not as readily apparent in Alternative Uses responding, though there was some relationship between inter-response time (IRT) and human rated similarity. However, since alternative uses responding relies to some extent on known associations, semantic similarity should be more strongly related to IRT in that task compared with the consequences task. This prediction is more tentative since it is plausible that both analyses show a weak relationship between IRT and semantic similarity, but the prediction is consistent with the prior research on the differential contributions of episodic memory to the two tasks.

The above logic is dependent upon the type of instructions used in the tasks. Two recent analyses showed that instructions to “be creative” while generating divergent thinking responses (as opposed to instructions to “think of as many responses as possible”) leads to lower output totals (Nusbaum et al., 2014; Forthmann et al., 2016), but higher creativity ratings. The boost in creativity is moderated by fluid intelligence, with more intelligent seemingly being better able to jump to more “creative” strategies (Nusbaum et al., 2014) throughout the task. In addition, the number of associations afforded by each DT prompt word (indexed by word-frequency) affected fluency, and to a lesser extent creativity and interacted with instruction type (Forthmann et al., 2016). So clearly, the type of instructions given to participants affects the kinds of memory processes in question here. In this paper, we opted to provide a middle ground between “be-creative” and “be-fluent” instructions because we used a 3-min time limit, but wanted to elicit an adequate number of responses per person for the purposes of evaluating the negative-exponential model of recall. This decision impacts the interpretation of our results and will be discussed later.

The novel components of this study include the various methods used to probe the predictions described above, which are not commonly applied to DT data. In addition, a web application was created to obtain the data. The app, which will be described in the section 2, relies on software created by the psiTurk project (McDonnell et al., 2012), a free and open-source set of python code that allows for experimental data to be collected in a controlled manner using participants recruited from MTurk. As will be described, the app and several helper functions are freely available to be adapted for use and can be downloaded from OSF and from the psiTurk experiment exchange (via github). The novelty of this component is that it allows researchers that may lack on-campus labs and participant pools to obtain reliable data regarding the cognitive processes involved in creative idea generation. The psiTurk code acts as an interface between user-generated HTML and JavaScript code and the MTurk platform, and several helpful features of that code enable controls on participants' workflow. In addition, the psiTurk code allows for data management and storage without the usual databasing infrastructure overhead that is needed for other apps and web interfaces. In this way, the novelty of the web app pertains to its ability to provide tools to small labs and independent researchers that might not otherwise be available to them.

## 2. Methods

### 2.1. Participants

Seventy-two participants (49 females) were recruited from MTurk. Participants were paid $2 US for successful completion of the experiment (i.e., accepting the HIT on Mturk, and proceeding through the entire experiment). Ages ranged from 19 to 69 years (*M* = 38.96, *SD* = 12.29) and 79% of the participants were caucasian (8% African American, 4% Hispanic/Latino, 9% Other). All participants consented to participate electronically, and the experimental procedure was approved by the first author's Institutional Review Board.

### 2.2. Materials

The experimental materials consisted of the experimental web-app, coded in JavaScript, the HTML pages that supported other parts of the experiment, and the supporting Python code that interfaced with MTurk. All are available via the psiTurk experiment exchange (http://psiturk.org/ee/PaY8pUQXu2yd2wraXHEiLA). Information and tutorials about the process of creating an experiment using psiTurk are available at http://psiturk.org.

#### 2.2.1. Physical features of the psiturk app

The web-app was written in JavaScript, and was laid out similar to a Matlab experiment used in previous studies (e.g., Hass, 2017a). Main instruction pages were presented to the participant along with specific instruction pages that preceded the two experimental blocks (one for alternative uses and the other for consequences), all with adequate font size. While responding, the cue was present on the screen in large font, and underneath the cue was a response field (an HTML text-entry field) labeled with the following text: “type responses here; press ENTER after EACH response.” Participants had full control of the response field with their keyboard and could use the backspace button to edit a response before pressing enter. When ENTER was pressed, the response field cleared so that the next response could be entered. When all tasks were completed, a survey page appeared with questions about age, sex, ethnicity, and a rating scale for engagement in the task (1 = not at all engaging; 10 = very engaging). A submit button appeared at the bottom of the survey page, which submitted the work to MTurk, and thanked the participant for participating.

In addition to collecting information about the type of browser the participant was using, when each browser event occurred (e.g., pressing a submit button), the main experimental data of interest were collected via the text-entry field. JavaScript functions were implemented to record the elapsed time between the presentation of the prompt and the first keypress for each response (response time), the latency between the first keypress of a response and the pressing of ENTER (entry time), and the actual text typed (response). Response time and the actual responses served as the primary data for analysis. Entry time was retained but not analyzed for this study.

#### 2.2.2. Creativity and similarity ratings

In addition to the response times collected via the app, 3 independent sets of ratings were obtained for the responses participants entered. Two raters were recruited from MTurk following the procedure detailed by Hass et al. (2018). Raters were supplied with two 5-point semantic differential scales, one created for Alternative Uses responses, and the other created for Consequences responses. As described by Hass and colleagues, the wording of the semantic differentials was created to assess how creative the responses were vis a vis the process by which the responses were generated. Raters were supplied with spreadsheets, one per prompt, and assigned a rating to each unique response from each prompt. Inter-rater reliability was evaluated using the intra-class coefficient, with guidelines for interpretation supplied by Cicchetti (2001). The inter-rater reliability estimates for Alternative Uses prompts ranged from fair to good (Brick ICC(2,2) = 0.50, Hammer ICC(2,2) = 0.70, Car Tire ICC(2,2) = 0.49). Inter-rater reliability estimates from Consequences prompts were generally fair (No Gravity ICC(2,2) = 0.52, 12-Inches ICC(2,2) = 0.48, No Sleep ICC(2,2) = 0.49).

A separate set of two raters provided ratings of similarity on a 4-point semantic differential (Hass, 2017a). These raters were not recruited from MTurk, but were undergraduate research assistants at the first author's institution. The raters were supplied with spreadsheets that gave the order of response, the participant who generated the response, and a blank cell to indicate the similarity between each pair of successive responses per participant per prompt. These raters achieved good to excellent reliability (Brick ICC(2,2) = 0.77, Hammer ICC(2,2) = 0.77, Car Tire ICC(2,2) = 0.85; No Gravity ICC(2,2) = 0.68, 12-Inches ICC(2,2) = 0.81, No Sleep ICC(2,2) = 0.79).

### 2.3. Procedure

MTurk is a service where “human intelligence tasks” (HITs) are posted with descriptions and an offer of payment. The psiTurk command line interface allows for posting batches of HITs, which appear on MTurk as ads. When a participant clicks on a HIT, a brief description is presented. For this experiment, the description advertised that this was an experiment about creative thinking in which there were going to think of creative ideas for six different prompts. They were also told that the experiment should last about 30 min. Once a participant “accepted” the HIT (meaning that he or she intended to participate), he or she was allotted 60 min to actually complete the experiment. Generally, if MTurkers participants do not leave enough time to finish HITs, they are free to “release” them for another MTurker to accept. Sixty minutes was more than enough time for participants to accept and complete the hit, and only two people failed to submit their work within the 60 min time period, both of which waited too long between accepting the HIT and beginning the experiment.

Immediately upon beginning the HIT, a pop-up appeared in a participant's browser containing a consent form, which could also be printed. To give consent, participants simply clicked “I agree,” and the experiment was launched. Two general instruction pages were then loaded, the first screen explained that there would be 6 experimental trials lasting 3 min each, and a practice trial lasting 30 s. The second screen explained that the experiment required them to type on their keyboard, and that the experiment would be split into 3 blocks, the 30-s practice block, and two 9-min experimental blocks. They were told that they could take short breaks between blocks, but reminded that they must finish the HIT within the allotted time.

Each block, including the practice block, contained additional instructions specific to the task. For the practice block, instructions were provided about the experimental interface, that it would contain a cue and a response field where they were to continue to type responses until the cue changed. They were told that the practice block was simply designed to orient them to the use of the response field. The practice prompt was to type “all the colors [you] know.” Participants were reminded in the instructions, and on the text-entry page to type enter after each response, and to keep thinking of responses for the entire time. A START button was visible on the bottom of the instruction page, and clicking it began the practice trial.

At the end of the practice block, and each subsequent block, the prompt field was cleared from the screen and a message appeared for 5 s, stating, “Good job! The next task is loading, please wait.” Another instruction page appeared for each experimental block, and participants were told that they could take a short break, but reminded that the HIT would expire in 60 min. The experiment did not proceed until the participant read the instructions for the block and clicked a START button on the bottom.

The order of the experimental blocks was counterbalanced: half of the participants began with the Alternative Uses prompts, and the other half began with the Consequences prompts. Within each block, the order of the prompts were randomized by JavaScript. The instructions for the Alternative Uses block read:

In the next set of tasks, the goal is to think of uses for objects. Please be as creative as you like. When you press Start, the name of a that object will appear on the screen. As soon as you think of something, type it into the field and press ENTER. Do this as many times as you can in 3 min. After 3 min on one category, the prompt will change to a new category, and after the next 3 min a third category will appear. This phase will last 9 min. Remember, it is important to try to keep thinking of responses and to type them in for the entire time for each prompt. Please type them in one at a time as they come to you, and press enter after entering each one.

The instructions for the Consequences block was similar, with the following change: participants were told that “a statement will appear on the screen. The statement might be something like *imagine that humans walked with their hands*. For 3 min, try to think of any and all consequences that might result from the statement. Please be as creative as you like.”

The prompts for the Alternative Uses task were brick, hammer, and car tire, and the prompts for the Consequences task were to imagine the consequences of “humans no longer needing sleep,” “humans becoming 12 inches tall,” and “gravity ceasing to exist.” On the text-entry page for Alternative Uses prompts, the text read “How can you use a(n) *OBJECT*?” to remind the participants that they must generate uses, not just associates for the object. On the text-entry page for Consequences prompts, the text read “What would happen if *SCENARIO*?”, again to remind them to generate consequences. In each case, the name of the object, or the scenario appeared in capital letters. Custom JavaScript functions recorded the response time (initial keypress), entry time (latency between initial keypress and pressing ENTER), and the actual text of each response. Data were saved to a dynamic MySQL instance hosted on Amazon Web Services, and parsed using a set of customized R functions which are downloadable here.

Prompts remained on the screen for 3 min, and were separated by a 5-s break, in which the prompt field cleared as well as the text-entry box, and a message stated “Good job! The next prompt is loading.” At the end of the final experimental block, the screen again displayed the “Good job” message, and the post-experiment questionnaire. Participants indicated their responses to questions about age, gender, ethnicity, and task engagement using drop-down menus. When they were finished, they pressed the submit button, and a thank-you message appeared on the screen. They were then directed back to MTurk, and received payment when the batch of HITs was completed and approved. All participants that submitted results successfully back to MTurk were paid, regardless of whether they completed the task correctly. Inspection of the data revealed one instance of an error in the logging of responses and 4 instances of participants neglecting to press ENTER to log responses. Thus, the final sample size was 67 participants.

## 3. Results

All data and analysis scripts and functions are available via the first author's Open Science Framework (osf.io/eux2k). Data parsing and analysis was performed using the R Statistical Programming Language (R Core Team, 2016), including the following packages: psych (Revelle, 2017), RMySQL (Ooms et al., 2017), jsonlite (Ooms, 2014), dplyr (Wickham et al., 2017), lme4 (Bates et al., 2015), lattice (Sarkar, 2008), and ggplot2 (Wickham, 2009). In all sections below, response times (RTs) represented the time between the presentation of the prompt and the time of the initial keypress leading to each response. This is consistent with recall studies that use voice-key technology to record response times, which are then defined according to the time of the initial voice onset of each response (e.g., Rohrer et al., 1995).

Four statistical analyses were planned: a descriptive analysis of the relationship between cumulative response counts and elapsed time (cumulative RT), a test of the difference in time to the first response (initial RT) across the two prompt types, a test of whether the relationship between pairwise similarity and inter-response time (IRT) differed by prompt-type, and a test of whether the serial order effect varied by prompt type. To aid interpretation of these analyses, descriptive statistics for fluency across the 6 prompts are listed in Table 1. Notably, fluency was, on average, significantly larger for Alternative Uses prompts (*M* = 9.68, *SD* = 4.01) than for Consequences prompts (*M* = 8.39, *SD* = 3.49), *t*_(66)_ = 3.44, *p* = 0.00, *d* = 0.42.

**Table 1:** Descriptive statistics for fluency (number of responses) across prompts (AU, Alternative Uses; C, Consequences, see text for full description of prompts).

**Prompt**	**Mean**	***SD***	**Median**	**Skew**	**Kurtosis**
Brick (AU)	10.12	4.52	10	0.86	0.71
Hammer (AU)	9.52	4.36	9	0.73	−0.07
Car Tire (AU)	9.39	4.37	9	0.79	0.51
12-Inches (C)	8.42	3.60	8	0.50	−0.49
No Gravity (C)	7.91	3.83	7	1.00	1.27
No Sleep (C)	8.84	4.53	6	0.91	0.26

### 3.1. Cumulative response curves by prompt

**Figure 1:**
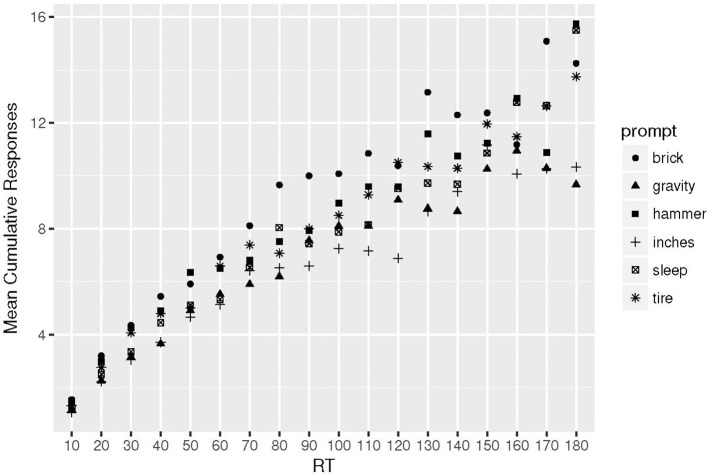
Plot of the Mean number of cumulative responses in successive 10 s blocks. Alternative Uses prompts: brick, hammer, tire; Consequences prompts: gravity, inches, sleep (see section 2 for full description).

The purpose of this is to examine whether there are differences in the cumulative response function across tasks. In Figure 1 plots of the average number of responses given by participants across successive 10-s blocks are shown. The plot is imprecise, such that toward the end of the interval, some of the slower participants had generated few responses, which resulted in the fluctuations seen on the right hand side of the plot. However, the plot suggests that a negatively accelerating cumulative RT function should provide an adequate fit to individual data across the prompts.

The function that is often used to approximate the trends seen in Figure 1 is an exponential function, in which the cumulative number of responses at time *t* is a curvilinear function that flattens out (reaches an asymptote) as time grows. The function was first derived by Bousfield and Sedgewick (1944) and is given by:

(1) $$\begin{array}{r} R\left(t\right)=a*\left(1-e^{-\lambda t}\right) \end{array}$$

where *R*(*t*) is the cumulative number of responses at time *t* and *e* is the exponential function. The constant *a* represents the “asymptotic level of responding” or the total number of items available for retrieval (Bousfield and Sedgewick, 1944). The constant λ is the rate of the exponential decay (deceleration), and was parameterized in terms of the inverse relation $\lambda =\frac{1}{\tau }$. In this parameterization, τ is the theoretical mean response time, which is a more interpretable parameter than λ in this context. Though Wixted and Rohrer (1994) suggested that τ can provide an index of search set size, here, mathematically, a larger τ represents a more linear cumulative response function (Xu, 2017), which was of interest in this analysis. In both cases, a larger τ represents a smaller λ, and following Bousfield and Sedgewick (1944) the equation represents the proportion of to be retrieved items left to be sampled at time *t* (see also Gruenewald and Lockhead, 1980).

Following from earlier work (Hass, 2017a; Xu, 2017), nonlinear least squares estimates of the asymptote (*a*) and mean response time (τ) were obtained for each participant using the “nls” function in R. Table 2 gives the quartiles of these estimates, for each prompt. Participants with fewer than 3 responses per prompt were excluded, but only for that prompt. In addition, as Table 2 illustrates, a few additional participants' estimates were not returned due to failure of the nls algorithm to converge. The results in Table 2 are consistent with Figure 1, in that the largest of the estimates of the τ parameter came from RTs for the Consequences of being 12 inches tall prompt. The results also illustrate that the exponential model predicts higher theoretical totals for fluency for the Consequences prompts than were actually observed (Table 1). These results are all consistent with Consequences prompts producing more linear cumulative response curves than Alternative Uses prompts.

**Table 2:** Median, Q1 and Q3 for the nonlinear least-squares estimates of asymptotic responding level (*a*), mean response time (τ) across prompts (AU, Alternative Uses; C, Consequences, see text for full description of prompts).

**Prompt**	***n***	***a*** **estimates**			**τ** **estimates**		
		**Q1**	**Median**	**Q3**	**Q1**	**Median**	**Q3**
Brick (AU)	63	7.85	10.80	16.72	31.72	51.24	86.01
Hammer (AU)	59	7.97	10.80	16.20	33.63	64.05	107.98
Car Tire (AU)	59	8.17	11.24	18.50	38.26	65.53	121.26
12-Inches (C)	57	8.58	12.04	18.73	66.97	102.90	172.05
No Gravity (C)	59	7.40	10.03	14.81	41.97	77.68	143.89
No Sleep (C)	56	6.97	10.13	18.61	51.08	87.78	164.49

*The scale of a is number of responses, whereas τ is reported in seconds*.

A statistical test for the last assertion is difficult to perform because due to the nature of nonlinear least squares estimates. However, a statistical test of the difference among the various response curves is possible using the discretized data that were the basis for Figure 1 in a mixed-effects regression model. The dependent variable in this model is cumulative responding with a discrete, integer predictor indexing which of the 18 10-s bins a response was output. A quadratic term for time-bin was added to the model to approximate the curvature of the exponential function. To test for the variation in curve shapes, the model included a fixed-effect of prompt (coded as a treatment contrast with the Brick task as the baseline) along with a cross-level interaction between the quadratic time-bin and prompt. Random intercepts and slopes per participant per prompt were also modeled. The numeric results are given in Table 3. Not surprisingly, the coefficients for linear and quadratic time-bin were significant, along with contrasts for output total. Importantly, the interactions between prompt and the quadratic time-bin term were significant for the No Gravity and 12-Inches prompt, with negative coefficients illustrating that these curves had less pronounced quadratic components (i.e., they were more linear) than the Brick curve. The curve for the No Sleep prompt did not significantly differ in it's quadratic component. Thus, there is evidence that cumulative response times are more linear for 2 of the Consequences prompts compared to the Brick prompt.

**Table 3:** Results of the Mixed-effects regression model of the RT curves, with cumulative response total as the dependent variable and 10-s block number as the discrete RT variable.

**Fixed effects**	**Coefficient**	***t***	***p***
Intercept	0.72		
RT (discrete)	1.04	49.55	< 0.001
RT-quadratic	−0.02	−12.48	< 0.001
No Gravity	−1.16	−4.58	< 0.001
Hammer	−0.62	−2.35	0.022
12-Inches	−1.46	−5.49	< 0.001
No Sleep	−1.37	−4.99	< 0.001
Car Tire	−0.91	−3.19	0.002
No Gravity*RT-quadratic	−0.008	−6.71	< 0.001
Hammer*RT-quadratic	−0.003	−2.69	0.007
12-Inches*RT-quadratic	−0.007	−6.41	< 0.001
No Sleep*RT-quadratic	−0.002	−1.88	0.060
Car Tire*RT-quadratic	−0.002	−1.71	0.087
**Random effects**	**Variance**		
Participant	2.88		
No Gravity	3.40		
Hammer	3.81		
12-Inches	3.86		
No Sleep	4.16		
Car Tire	4.47		
Residual	2.20		

*The baseline level for the contrasts was the Brick prompt*.

### 3.2. First response latency by condition

As an additional test of the processing differences between Alternative Uses and Consequences items, the RTs for first responses on the three Alternative Uses prompts were averaged, as were the RTs for the first responses to the 3 Consequences prompts. This seemed feasible given the results above, that all 6 prompts are well approximated by the exponential function, with varying parameters. The RT averages were skewed, due mainly to a few participants who took a long time to begin responding (which was later found to be a flaw in the design of the app). To test for a difference in initial RT across the tasks, without an assumption of normality, a Wilcoxon signed rank test (with continuity correction) was performed in R. The test was significant such that initial RTs were shorter for the Alternative Uses prompts than for the consequences prompts, *z* = −2.16, *p* = 0.03, *r* = −0.26. The effect size is small to medium using Cohen's (1988) guidelines for effect size *r* (Fritz et al., 2012), suggesting that there may be a small increase in initial processing involved when generating responses to Consequences prompts.

### 3.3. Pairwise similarity by prompt

The third planned analysis examined the relationship between pairwise similarity and IRT. Theoretically, if the Alternative Uses task involves searching through a memory store that is more highly clustered, there should be a stronger relationship between pairwise similarity and IRT for those prompts compared with Consequences prompts. That is, theoretically, short IRTs would indicate less remote association between successive responses. The consequences task, which may depend only on semantic memory, and also on other reasoning processes, should theoretically have a looser relationship between IRT and pairwise similarity. That hypothesis was tested by fitting a linear mixed effects model, with pairwise similarity rating as the dependent variable, IRT as a level-1 independent variable, and prompt-type (Uses v. Consequences) as a level-2 variable (fixed effect). Random intercepts for prompt (all 6 levels) and participant were included in the model to account for the repeated measures nature of the design. Modeling a cross-over interaction between prompt-type and IRT did not improve the fit of this model [$\chi_{\left(1\right)}^{2}=0.20$, *p* = 0.65], meaning that there was no significant difference in the slope of the IRT - similarity relationship across the two prompt types.

**Figure 2:**
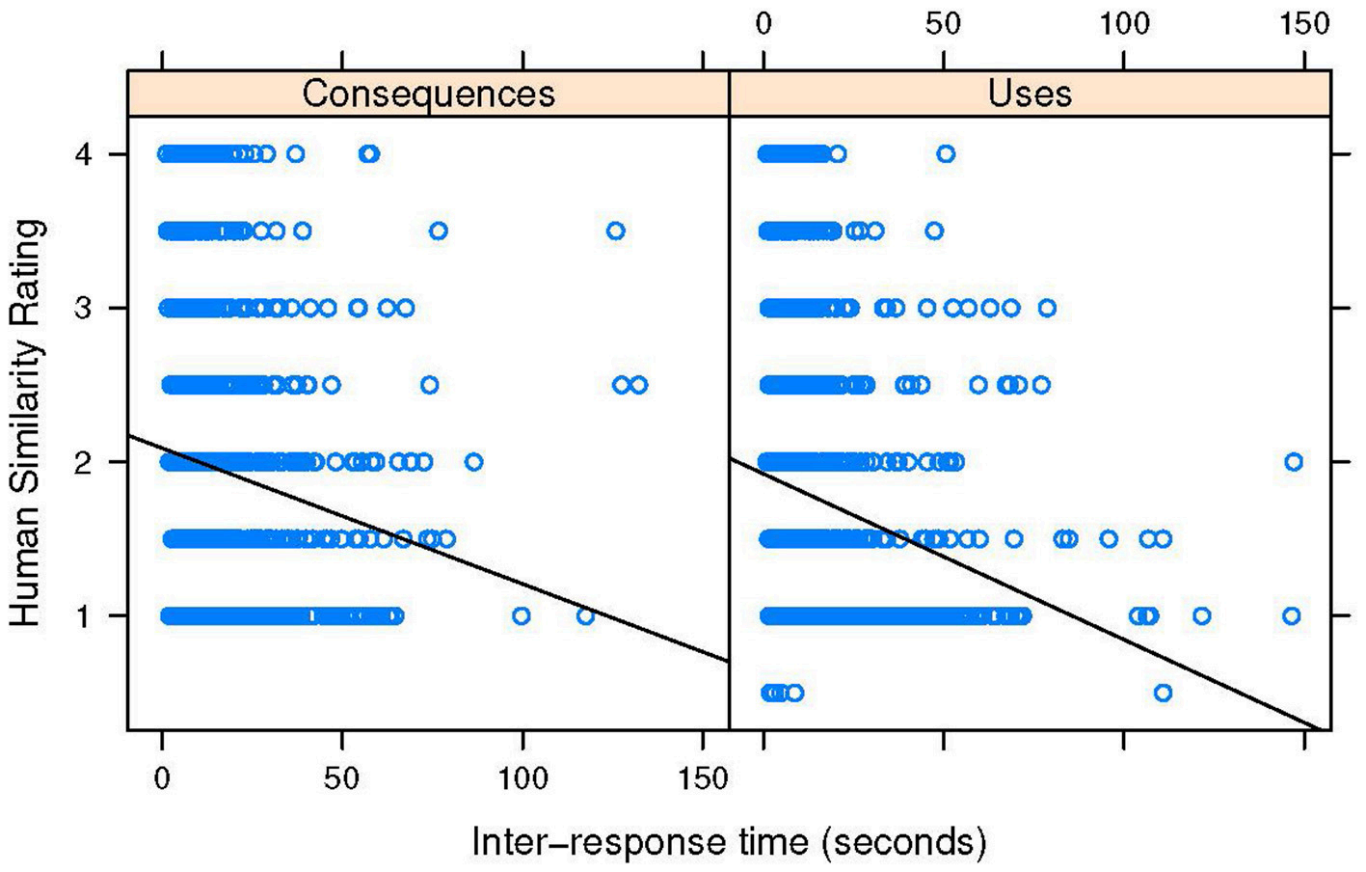
Scatter plots of inter-response time and pairwise similarity for the two prompt conditions. Solid line represents ordinary least squares regression. See Table 3 for the actual regression results from mixed-effects modeling.

Table 4 contains the full results of the model with no interaction term. The fixed effect of condition was not significant indicating a non-significant difference in average pairwise similarity across the two prompt types. However, the IRT - similarity association was significant, such that as IRTs increased, pairwise similarity tended to decrease. Figure 2 illustrates these trends, and also shows that indeed, there seems to be little difference in the IRT-similarity slopes. However, the Figure also illustrates a clear nonlinear pattern in the results: short IRTs show a variety of different pairwise similarity values, but as IRTs increased, similarity decreases. Indeed, a quantile-quantile plot of residuals suggested that the model over-predicts pairwise similarity for short IRTs, and under-predicts pairwise similarity for long IRTs. So a more conservative conclusion is that the relationship between IRT and pairwise similarity does not systematically vary by prompt-type, and that the linearity of the relationship may be overstated by the model. Contrary to the hypothesis, the two tasks seem to show the same degree of relationship between IRT and pairwise similarity.

**Table 4:** Results of the Mixed-effects regression model with pairwise similarity as the dependent variable.

**Fixed effects**	**Coefficient**	***t***	***p***
Intercept	2.12	18.260	
IRT	−0.01	−8.50	< 0.001
Prompt-type	−0.20	−1.30	0.25
**Random effects**	**Variance**		
Participant	0.038		
Prompt	0.036		
Residual	0.784		

*Consequences was the baseline Prompt-Type. The random effect of Prompt is the variance component across all 6 prompts*.

### 3.4. Serial order by task

The final question asked in this analysis was whether the serial order effect varied by prompt type. Again, a mixed-effects model was fit, this time with creativity ratings as the dependent variable, the order of the response as the level-1 predictor, and a level-2 predictor for prompt-type (Alternative uses vs. Consequences). Response order was rescaled with the first response denoted by 0. To remove the potential of outliers (highly fluent individuals) to affect these results, serial order analysis was limited to the first 14 responses. This value was chosen because 95% of participants gave 14 or fewer responses on the consequences prompts. The 95th percentile of fluency for the Alternative uses prompts was around 17. To make this analyses equitable, the smaller of the two values was chosen.

**Figure 3:**
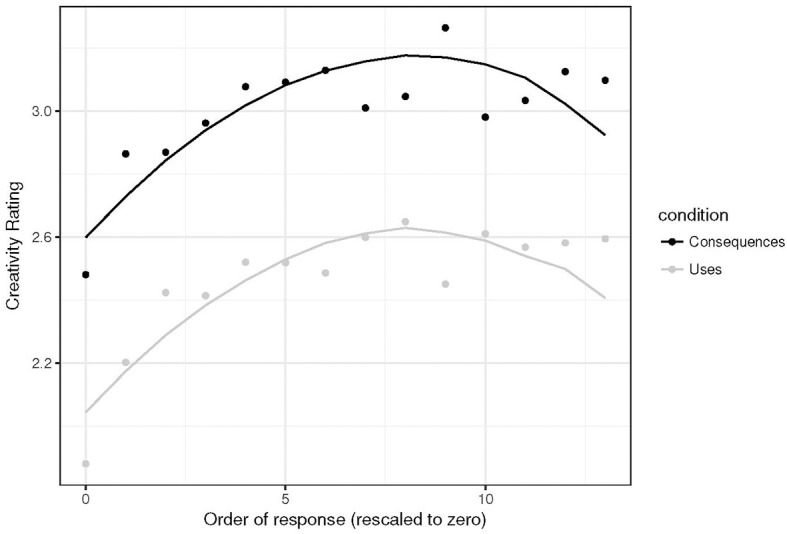
Data (dots) and model predictions (line) for the serial order effects across the two prompt types. Note that the order of responses was re-scaled with 0 as the first response.

Again, random intercepts for prompt-type and participant were included to model the repeated measures nature of the design. Following Beaty and Silvia (2012), both linear and quadratic order effects were modeled. An interaction between prompt type and the linear serial order term did not improve the model fit [$\chi_{\left(1\right)}^{2}=1.04$, *p* = 0.31], nor did a quadratic serial order term improve the fit [$\chi_{\left(1\right)}^{2}=0.86$, *p* = 0.35]. So the best model was that including a linear serial order term and a fixed-effect of prompt type, along with the random intercepts described above. The quantile-quantile plot of the residuals from this model suggested that the residuals did conform to normality, unlike the IRT model. Table 5 contains the full output from the final model. There are significant linear and quadratic trends, which replicates the results of earlier serial order effects analyses (Beaty and Silvia, 2012). In addition, the ratings from Alternative Uses tasks were significantly lower at the onset of responding, but with no interaction, Figure 3 illustrates that serial order effects are the same, albeit offset for the two prompt types. As such, it seems that participants begin with more creative responses to the Consequences prompts compared to the Alternative Uses prompts, but that the serial order effect remains in tact for both prompt types.

**Table 5:** Results of the Mixed-effects regression model of the serial order effect (Creativity as the dependent variable).

**Fixed effects**	**Coefficient**	***t***	***p***
Intercept	2.598		
Order (linear)	0.139	12.93	< 0.001
Order (quadratic)	−0.008	-8.94	< 0.001
Prompt-type	−0.554	−4.18	0.006
**Random effects**	**Variance**	
Participant	0.026	
Prompt	0.026	
Residual	0.456	

*Consequences was the baseline Prompt-Type. The random effect of Prompt is the variance component across all 6 prompts*.

## 4. Discussion

The present study was motivated by theoretical and practical issues. The theoretical motivations will be discussed first in light of the data. To address a hole in the burgeoning research on memory processes in creative thinking, the Alternative Uses task was compared to the Consequences task using a variety of metrics derived from existing analyses of memory retrieval. A recent analysis (Madore et al., 2016) suggested that the Consequences task may be less dependent on well-learned episodic information, and the results of this analysis are consistent with that interpretation. First, in Figure 1, the rate of exponential growth of cumulative responses on the consequences prompts was, on average, slower. This can be seen for example, by examining the points where *t* = 60. There are clearly two clusters of points, the bottom of which consist of the mean cumulative number of responses for the 3 consequences prompts, which appear nearly 2 units lower than the three points representing the 3 alternative uses prompts. The separation between these points begins around 20 s, and is clear through about 70 s, where the mean cumulative responses become more variable. The individual fits of the exponential response time function in Table 2 confirm along with the regression analysis that for at least 2 of the consequences prompts, output was more linear. This is consistent with Xu's results that when participants are instructed only to generate “new” Alternative Uses for objects in a creative task, the rate of exponential growth (1/τ) of the response time function is slower, as it was here for Consequences responding. Xu also showed that when constraining participants to think of only “new” uses, their output totals are smaller, which is again consistent with the current analysis.

The slower rate of responding may be a function of additional processes operating during the Consequences task, such the initial time to respond to consequences prompts was significantly longer than the initial response time for alternative uses prompts. This suggests that either the encoding of the cue and initial search of memory takes longer for consequences prompts, or that in addition to encoding the cue and searching memory, consequences responding requires additional cognitive processing to continue. Unfortunately, the current analysis could not disentangle encoding from additional processes, but it is likely that future behavioral or neuroscientific studies will be able to do so. As mentioned, one candidate process involves counterfactual reasoning about the impossible events represented by consequences prompts. However, it is also likely that the Consequences tasks are executively more demanding, and that it is an executive slow-down that is occurring, rather than a superposition of memory and reasoning processes.

Finally, the results of the serial order analysis provide further evidence that during consequences generation, participants are better able to generate more creative responses from the beginning of the response interval. However, there was still a serial order effect for Consequences prompts, meaning that remote association may form the core of Consequences generation, as it does for Alternative Uses generation. That interpretation is supported by the lack of a difference in the relationship between inter response time (IRT) and pairwise similarity across the two types of prompts. Though the IRT-similarity relationship does not seem to be linear, the amount of pairwise similarity did not vary significantly across the two types of prompts. This suggests that either the type of knowledge accessed during generation of both types of ideas is not likely to be strongly associated with other knowledge to the task, or that some executive process intervenes to override local cues during the generation of creative responses (cf. Troyer et al., 1997; Hills et al., 2012; Hass, 2017a). An answer to that question rests upon further analysis of the existence of semantic clusters of responses in these arrays, which is beyond the scope of the current study. Indeed, while norms exist to identify clusters in semantic categories such as animals (Troyer et al., 1997) there are currently no published norms for Alternative Uses responses, and norms for Consequences responses are proprietary. Though many researchers use their own systems for categorizing DT responses (for the purposes of flexibility scoring), a normative system for such categorization would be helpful to further probe the regularities of the search process involved by enabling more thorough computational modeling of idea generation.

### 4.1. Implications for further cognitive and neuroscientific studies

In the introduction, several pieces of new research pointing to a specific set of cortical structures within the default network supporting creative thinking were reviewed. Because this network shows reliable activation during tasks involving episodic retrieval and simulation (Gerlach et al., 2011), it has been hypothesized that activation of these regions in studies of creative thinking reflect the involvement of episodic retrieval mechanisms (Addis et al., 2016; Madore et al., 2017). Perhaps the clearest evidence for a role of episodic retrieval comes from Madore et al. (2017), who found that an episodic specificity induction boosted performance on the alternate uses task, which corresponded to increased activity within the left anterior hippocampus of the default network. Another recent study by Benedek et al. (2018) found that default network regions (hippocampus and medial prefrontal cortex) are involved in both the recall of original object uses and the imagination of novel object uses (i.e., the generation of “old” and “new” ideas, respectively; Benedek et al., 2014) compared to a control task that does not require creative thinking. Contrasting old and new idea generation directly, however, revealed selective engagement of the left supramarginal gyrus (SMG) during the generation of new ideas (Benedek et al., 2014, 2018). In light of the SMG's role in cognitive control processes and constrained memory retrieval, Benedek and colleagues hypothesized that the generation of new ideas involves more executively-demanding mental simulations that are less relevant for the retrieval of old ideas from episodic memory. Critically, however, neuroimaging work has largely focused on the Alternate Uses task, so the extent to which similar brain regions are involved in Consequences generation remains an open question. Taken together with recent behavioral and neuroimaging work on old and new ideas, the current results suggest that, because Consequences responses tend to be more “new” than “old,” one might expect executive brain regions to come online to support such complex search and retrieval processes. This is among the speculations relayed previously about the Consequences task, and the current results suggest that it may be advantageous to begin to compare the uses and consequences prompts in the scanner.

### 4.2. Practical considerations for using MTurk and psiturk

Despite the success of this project, and the building of a useable interface to conduct these kinds of experiments using MTurk workers, there are a few practical issues to consider in follow-up studies. First, MTurkers are very sensitive to the directions. In pilot testing, participants tended to not press ENTER unless explicitly instructed to do so on the text-entry page, meaning data were lost. The current app includes instructions which are very specific and repeatedly remind the participant to press enter and to continue thinking of responses. Even so, at least 1 participant per prompt exhibited atypical initial response times (e.g., initial RT > 30 s), which may be due to distraction. Though the age range of participants on MTurk is larger than that for normal laboratory based psychology experiments, using MTurk successfully, and getting work approved usually requires that people are computer savy. That is, the small number of long latencies is not expected to be a function of the age of the participants. Even though one participant reported being 69 year old, a majority of participants were between the ages of 24 and 50 year old. The relationship between age and initial latency was not tested, however, and may be a relevant research question for future studies.

The app, as it is now constructed, does not allow the participant to take an extended break within an experimental block, only between blocks. Within a block, the prompt would change after 3 min plus a 5 s delay. If the participant became distracted, there was no way for him or her to notice the fact that the next task started, and latencies were biased by the distraction. Again, this was rare, but the fact that it happened more than once means that initial steps must be taken to control the flow of the program, or to set exclusion criteria. Since exclusion criteria set prior to the experiment were simply designed to filter out participants who did not follow directions or who did not respond to all tasks, it was decided that these atypical latencies should be retained for transparency. Due to the nature of the analysis, these latencies did not greatly affect the results, but the app has been updated to include a button press (space-bar) between each prompt presentation, so that MTurkers can move at their own pace.

### 4.3. Theoretical limitations and alternative explanations

Aside from practical considerations, a few limitations and alternative explanations exist. First, the residuals of the linear mixed-effects regression of similarity on IRT and prompt type violated the assumption of normal residuals, and the model may be overshooting the relationship between IRT and pairwise similarity. As mentioned, the lack of norms for responses on both kinds of prompts used in this study makes other IRT analyses difficult, but such analyses are necessary before firmer conclusions are made about the IRT-similarity relationship during creative thinking tasks. As an alternative, ordinal multilevel regression models could be used, as the similarity scale can be treated as ordinal. For example, Forthmann et al. (2016) used linear response trees to examine the interaction of word frequency and be-creative instructions with very interpretable results.

In addition, there may be an alternative explanation for the difference in creativity ratings between Alternative Uses and Consequences responses. The rating scales used to rate the responses do differ with respect to the scoring criteria in a nature relevant to this difference in response length. For consequences responses, the maximum creativity rating for consequences responses (5 out of 5) is “very imaginative/detailed consequence”, while the maximum creativity rating for uses is “very imaginative / re-contextualized use.” The rationale for the difference between the two is that the scores are then specific to the goals of the tasks. In constructing those scales, it was reasoned that a very creative consequence should be one in which a detailed thought process was carried out. However, Consequences responses may simply earn higher ratings because they contain a greater number of words on average (Forthmann et al., 2017). This issue of scoring differences across creative thinking tasks is at the heart of a larger debate in creativity about domain-specificity (Baer, 2011). That said, the participants were not aware of the criteria used for rating their responses, and are not explicitly told to be detailed in the responses to either task. So from that perspective, the fact that Consequences responses tend to be longer seems to be related to the nature of the prompt rather than an artifact of the rating procedure. Still more research into the reasoning processes that underpin the Consequences task is necessary to shed more light on this issue.

Finally, the instructions given to participants deviated from the “be creative” instructions used in more recent studies. The choice was made to use the current instructions in order to facilitate higher fluency totals. This, however, is a limitation of the method, as it can be argued that instructing participants to “be creative as [they] like[d]” leaves open the question as to whether all participants interpreted the tasks in the same way. This is an important caveat, though the results are in line with predictions based on studies that used “be creative” instructions. It would be advantageous, however, to investigate whether the exponential parameters fit to RT data in the current study would change when “be creative” instructions are used, compared to “be fluent” instructions.

### 4.4. Concluding remarks

The goals of this study were both practical and theoretical. On the practical side, a workable web interface for collecting creative thinking data from MTurk workers is now available to the scientific research community. Moreover, the data generated by participants in the web environment are consistent with data generated in the lab (cf. Hass, 2015, 2017a), and can be used to test cognitive hypotheses. This is a novel development as it can allow for researchers with limited lab space and lack of participant pools to collect valuable data about cognitive processing in divergent thinking. In addition, since MTurkers represent a more wide-ranging demographic than undergraduate participant pools, the results may be more externally valid.

On the theoretical side, the results suggest that both Alternative Uses and Consequences tasks tap the same general processes, and conform to the serial order effect. This is a novel result as the serial order effect has never been explored with Consequences prompts. However, there were subtle differences such that the initial processing time for Consequences responding is slightly longer than Alternative Uses, and Consequences responses seem to earn higher creativity ratings from the start of the idea generation process. The latter effect may due to additional reasoning required to either search for or evaluate potential Consequences before they are output. Evidence from cumulative RT analysis provides some support for that assertion, but it is hoped that future research with computational models and brain imaging techniques will provide more insight. At the same time, both Alternative Uses and Consequences tasks can continue to be used as measures of divergent thinking.

## Ethics statement

This study was carried out in accordance with the recommendations of APA ethics guidelines for ethical treatment of participants. The protocol was approved by the Institutional Review Board, Thomas Jefferson University. All subjects gave written informed consent in accordance with the Declaration of Helsinki.

## Data availability statement

The datasets generated and analyzed for this study can be found on the Open Science Framework repository for **this study**.

## Author contributions

RH conceived of the study and ran the analyses. RH and RB wrote the paper.

### Conflict of interest statement

The authors declare that the research was conducted in the absence of any commercial or financial relationships that could be construed as a potential conflict of interest.
